# Cytotoxic and Antitumor Effects of the Hydroalcoholic Extract of *Tagetes erecta* in Lung Cancer Cells

**DOI:** 10.3390/molecules28207055

**Published:** 2023-10-12

**Authors:** Alma Sarahi Cuellar González, Marisol Galván Valencia, Rodolfo Daniel Cervantes-Villagrana, Alondra Bocanegra Zapata, Alberto Rafael Cervantes-Villagrana

**Affiliations:** 1 Maestría en Ciencia y Tecnología Química, Unidad Académica de Ciencias Químicas, Universidad Autónoma de Zacatecas, Zacatecas 98160, Mexico; 2 Laboratorio de Investigación en Patología y Productos Naturales, Unidad Académica de Ciencias Químicas, Universidad Autónoma de Zacatecas, Zacatecas 98160, Mexico; 3 Departamento de Farmacología, Centro de Investigación y de Estudios Avanzados del Instituto Politécnico Nacional (CINVESTAV-IPN), México City 07360, Mexico; 4 Laboratorio de Investigación en Terapéutica Experimental, Unidad Académica de Ciencias Químicas, Universidad Autónoma de Zacatecas, Zacatecas 98160, Mexico

**Keywords:** cancer, cytotoxicity, *Tagetes erecta*, lung cancer, plant extract

## Abstract

Among all cancers, lung cancer is the one with the highest mortality rate, and it also has limited therapeutics. Antitumor agents based on medicinal plants have gained importance as a source of bioactive substances. *Tagetes erecta* is a plant of great cultural value, and recent reports have suggested its cytotoxic effects in tumor cells. Our objective was to evaluate the antitumor activity of *Tagetes erecta* extract in a lung carcinoma model. Hydroalcoholic extracts were obtained from fresh flowers and leaves of *T. erecta*; both extracts did not exert toxicity on *Artemia salina*. We observed cytotoxic effects induced by the floral extract in Lewis lung carcinoma (LLC) and breast tumor cell line (MCF7), but not by the leaf extract. In vivo, a xenograft lung carcinoma model was performed with LLC cells implanted on C57BL/6 mice, which showed that the floral extract reduced tumor growth and improved the effect of etoposide. Microscopic analysis of tumors showed a reduction in mitoses and an increase in necrotic areas with the extract and the etoposide. The main phytochemical compounds found are 2,3-dihydro-benzofuran, octadecanoic acid, benzenacetic acid, oleic acid, linoleic acid, and acetic acid. We conclude that the hydroalcoholic extract of *T*. *erecta* flowers has cytotoxic effects in lung carcinoma cells and enhances the effect of etoposide.

## 1. Introduction

According to the 2020 estimates by GLOBOLCAN, cancer is the leading cause of death worldwide; every year, 19.3 million new cases are recorded, and it takes the lives of approximately 10 million people around the world. Among all cancers, lung cancer has the highest mortality rate in the general population, with an estimated 1.8 million deaths per year [1]. Although all oncological diseases are important, currently, the main concern lies with lung cancer (LC) because, unlike other cancers, it is characterized by higher aggressiveness and a higher mortality [1]. Initially, lung cancer cases were attributed almost entirely to smoking. However, in recent decades, evidence has emerged showing that, globally, 25% of cases occur in non-smokers, in whom a multifactorial etiology is considered, such as environmental, occupational, and genetic factors [2].

Currently, the available drugs for cancer treatment have limited therapeutic potential due to high toxicity, inefficiency, and high cost. Considering these facts, the development of treatments without these disadvantages is necessary. Therefore, the identification and synthesis of new efficient and less toxic anticancer agents remain an important and challenging task for cancer treatment. For this reason, natural products have become increasingly relevant in the pharmaceutical field, and traditional herbalism has been a pioneering specialty in biomedical sciences. The search for effective plant-derived anticancer agents has gained momentum in recent years. Conventional plants are a valuable source of new cytotoxic agents and play a critical role in health due to their wide range of biological and pharmacological effects and lack of toxicity [3,4,5,6].

Aztec Margaroid, scientifically named *Tagetes erecta*, is an annual, dicotyledonous herbal species that measures between 60 cm and 1 m in height, with aromatic characteristics. Its stems have small grooves, and it has opposite leaves with indentations with serrated edges [7]. In addition to its cultural and ornamental value, it is commonly used in the ethnobotanical field, and its properties have been considered to be useful in treating bronchitis, rheumatic pain, and respiratory diseases. *Tagetes erecta* has been used as a muscle stimulant and relaxant [8], as an anticancer agent, and also used in the treatment of gynecological problems [9]. Different studies have shown the cytotoxic effect of *Tagetes erecta* on cancer by evaluating its impact on several tumor cell lines such as B16F10 (murine melanoma), HT29 (human colon carcinoma), MCF-7 (human breast adenocarcinoma), HeLa (adenocarcinoma human cervical), HepG2 (human hepatocellular liver carcinoma), MO59J, U343 and U251 (human glioblastoma) [10,11]. Moreover, it has been described that *Tagetes erecta* extracts inhibit tyrosinase and elastase in the H-460 (lung cancer) and Caco-2 (colon cancer) cell lines [12]. The active compounds found in the extracts of *T. erecta* flowers and leaves are hexadecanoic acid, linoleic acid, quinic acid, coumaran, β-Stigmasterol, Limonene, (Z)-β-Ocimene, Terpinolene, (Z)-Ocimenone, (E)—Ocimenone and β-Caryophyllene [6,13]. However, there is little evidence on *T. erecta’s* cytotoxic effect in lung cancer cells and its antitumor effects in animal models. Therefore, the present research aims to evaluate the cytotoxic effect of *T. erecta’s* extract on the Lewis lung carcinoma cell line, as well as in a syngenic murine lung cancer model.

## 2. Results

### 2.1. Safety Evaluation of Hydroalcoholic Extracts of Tagetes erecta

**Figure 1:**
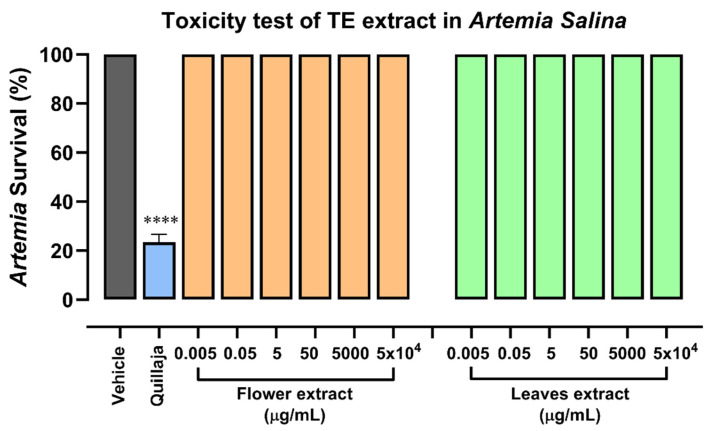
Toxicity test of *T. erecta* extracts in *Artemia salina*. Concentrations of 50 mg/mL, 5 mg/mL, 50 µg/mL, 5 µg/mL, 50 ng/mL, and 5 ng/mL of aqueous extracts of flowers and leaves were used for treating 10 nauplii of *Artemia salina* per tube, and the percentage of survival was calculated. A saline solution of 35 g/L was used as a negative control, and standard quillaja toxicity was used as a positive control. Error bars indicate the mean ± SEM of 3 independent experiments. One-way ANOVA followed by Dunnett’s post hoc was used; **** *p* < 0.0001 versus control (vehicle).

We obtained hydroalcoholic extracts from the flowers and leaves of *Tagetes erecta* and assessed their toxicity level using the *Artemia salina* model (Figure 1). The *Artemia* bioassay is useful for the biological evaluation of natural products since it is a method of rapid evaluation used as a substitution of superior animal models. In the exposition of *Artemia* to the extracts of flowers and leaves during 24 h, no toxic effect was observed for any extract, which could indicate that none of the extracts has a toxic effect on healthy organisms. In parallel, we included the evaluation of the quillaja effect as a positive control, where *Artemia* survival decreased to 20%; this result showed that the model worked in our conditions, and the extracts were not toxic.

### 2.2. T. erecta Extract Induces Cytotoxicity in Tumor Cell Lines

**Figure 2:**
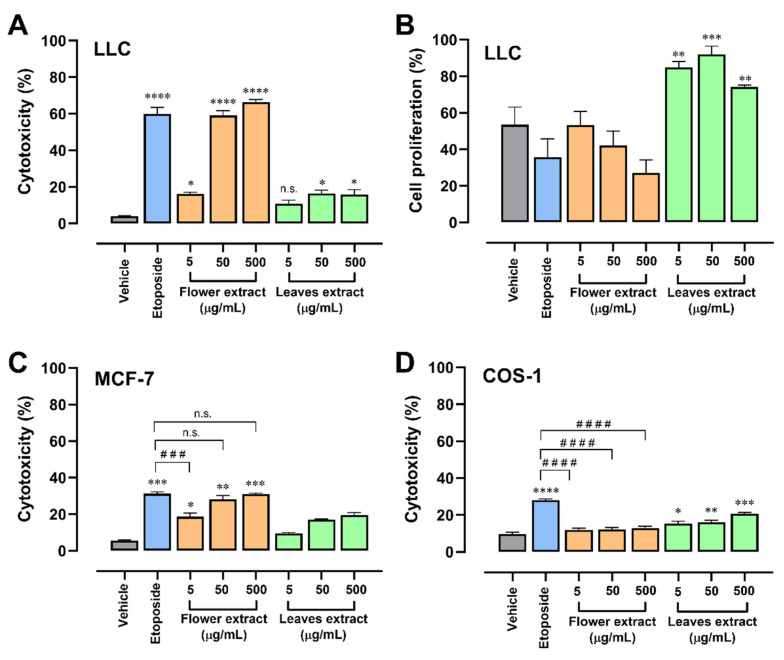
Floral extract of *Tagetes erecta* has a selective cytotoxic effect in cancer cells. (**A**) Cytotoxic effect of the hydroalcoholic extract of flowers and leaves of *Tagetes erecta* on the LLC tumor cell line. Supplemented DMEM was used as a negative control, and etoposide (50 µM) was used as a positive control. Different concentrations of the *T. erecta* extracts were evaluated after 24 h of treatment. One-way ANOVA followed by Dunnett’s post hoc was used. Error bars indicate the mean ± SEM of 3 independent experiments in triplicate. * *p* < 0.05, **** *p* < 0.0001, n.s. not significant, all versus negative control (vehicle). The results are expressed as a percentage of cytotoxicity. (**B**) The antiproliferative effect of the hydroalcoholic extract of flowers and leaves of *Tagetes erecta* in the LLC tumor cell line. Supplemented DMEM was used as a negative control, and etoposide (50 µM) was used as a positive control. The different concentrations were evaluated after 24 h of treatment. The data represent the mean ± SEM of 3 independent experiments in triplicate. One-way ANOVA followed by Dunnett’s post hoc was used. ** *p* < 0.01, *** *p* < 0.001 versus negative control (vehicle). Results are expressed as optical density, which is proportional to cell proliferation. (**C**) Cytotoxic effect of the hydroalcoholic extract of flowers and leaves of *Tagetes erecta* on the tumor cell line MCF-7. Supplemented DMEM was used as a negative control, and etoposide was used as a positive control. *T. erecta* extracts were evaluated after 24 h of treatment. Error bars indicate the standard deviation of 3 independent experiments in triplicate. One-way ANOVA with Dunnett’s post hoc was used; * *p* < 0.05, ** *p* < 0.01, *** *p* < 0.001 versus negative control (vehicle); ^###^ *p* < 0.001, n.s. not significant versus positive control (etoposide). The results are expressed as a percentage of cytotoxicity. (**D**) Cytotoxic effect of the hydroalcoholic extract of flowers and leaves of *Tagetes erecta* on the non-tumor cell line COS-1 (monkey kidney fibroblasts). Supplemented DMEM was used as a negative control, and etoposide was used as a positive control. Different concentrations of the *T. erecta* extracts were evaluated after 24 h of treatment. Error bars indicate the standard deviation of 3 independent experiments in triplicate. One-way ANOVA with Dunnett’s post hoc was used; * *p* < 0.05, ** *p* < 0.01, *** *p* < 0.001, **** *p* < 0.0001 versus negative control (vehicle); ^####^ *p* < 0.0001 versus positive control (etoposide). The results are expressed as a percentage of cytotoxicity.

The leaf and flower extracts were evaluated to select the extract with the best effect to continue with the following evaluations. Cell viability was evaluated using the trypan blue method after 24 h of treatment in LLC (Lewis lung carcinoma) cell lines. Both extracts were observed to exert a cytotoxic effect, but the leaf extract was less effective and that of the floral extract was comparable to an etoposide, which is an inhibitor of topoisomerase useful in lung cancer treatment [14]; we included it as a positive control (Figure 2A). Then, when the effects of the extracts were evaluated using a proliferation assay, it was seen that a high concentration of floral extract showed a decrease in the proliferation of the LLC cells (Figure 2B). The floral extract was the considered as the best, since the effect could be observed from the lowest concentration (5 µg/mL); this effect increased depending on the dose. In the case of the leaf extract, a ceiling effect could be observed at the concentration of 50 µg/mL; the effect exerted at the highest concentrations is comparable with the lowest concentration of the floral extract. For this reason, its use was ruled out for further experiments.

To evaluate the selectivity of the extract in the lung cancer cell line, both extracts were tested in MCF-7 cells (Figure 2C), a breast cancer cell line that had already been evaluated with the essential oil [10]. The cytotoxic effect of the floral extract increases in a dose-dependent manner. The concentration of 50 and 500 µg/mL had an effectivity comparable with etoposide (~30% cytotoxicity), while the leaf extract was weak and did not obtain a significant effect, which is consistent with the effect in LLC cells. In parallel, the effect of extracts was assessed in the non-tumor cell line COS-1 using different concentrations of both extracts for 24 h. In this experiment, we hypothesized that the floral extract had cytotoxic selectivity for cancer cells, without a cytotoxic effect on non-tumor cells (Figure 2D). None of the tested concentrations of floral extract induced cytotoxicity in non-tumor cells, which indicates that the extract has a lower toxicity in normal cells compared to cancer cells, showing selectivity to malignant cells. In contrast, the leaf extract showed significant cytotoxicity in non-tumor cells, and together with the results of low cytotoxic effectiveness in tumor cells and the induction of LLC proliferation, experimental evaluations of the leaf extract in the following trials were ruled out.

### 2.3. The Combination of T. erecta Extract and Etoposide Decreases Cell Proliferation

**Figure 3:**
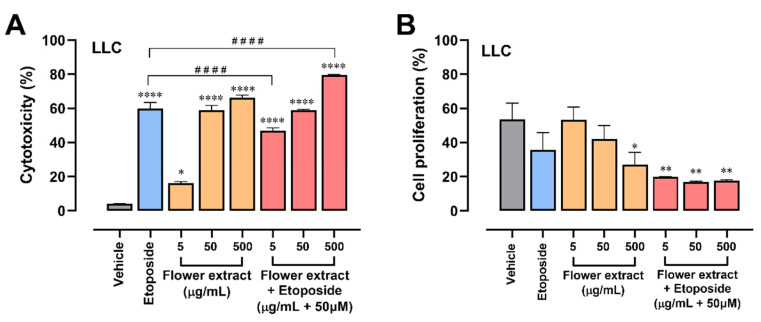
Floral extract of *T. erecta* enhances the cytotoxic effect of etoposide. (**A**) Cytotoxic effect of the hydroalcoholic extract of flowers of *Tagetes erecta* and combination of the extract with etoposide in the LLC tumor cell line. Supplemented DMEM was used as a negative control, and etoposide was used as a positive control. Different concentrations of the *T. erecta* extract were evaluated after 24 h of treatment using the trypan blue method. One-way ANOVA followed by Dunnett’s post hoc was used. The indicated significances were compared with the control group. Error bars indicate the mean ± SEM of 3 independent experiments in triplicate: * *p* < 0.05, **** *p* < 0.0001 versus vehicle; ^####^ *p* < 0.0001 versus positive control (etoposide). The results are expressed as a percentage of cytotoxicity. (**B**) The antiproliferative effect of the hydroalcoholic extract of flowers and leaves of *Tagetes erecta* in the LLC tumor cell line. Supplemented DMEM was used as a negative control and etoposide as a positive control. Different concentrations of the *T. erecta* extract were evaluated after 24 h of treatment. One-way ANOVA followed by Dunnett’s post hoc was used. Error bars indicate the mean ± SEM of 3 independent experiments in triplicate: * *p* < 0.05, ** *p* < 0.01 versus negative control (vehicle). Results are expressed as optical density, which is proportional to cell proliferation.

To evaluate whether the extract, in addition to being cytotoxic, had antiproliferative activity, the crystal violet method was used, where cell proliferation was evaluated after 24 h of treatment with different concentrations of both extracts. In Figure 3A, the extract did not show a significant decrease in cell proliferation; however, a trend can be observed at a concentration of 500 μg/mL. Contrary to what was expected, the leaf extract has a significant increase in cell proliferation compared to the control without treatment, another reason why its use was ruled out in future evaluations. As can be seen in Figure 3B, when combining the extract with 50 µM etoposide, a significant antiproliferative effect is reached in all the extract concentrations evaluated. This confirms the use of an etoposide-extract combination group for further evaluation.

### 2.4. Tagetes erecta Extract Inhibits Tumor Growth

**Figure 4:**
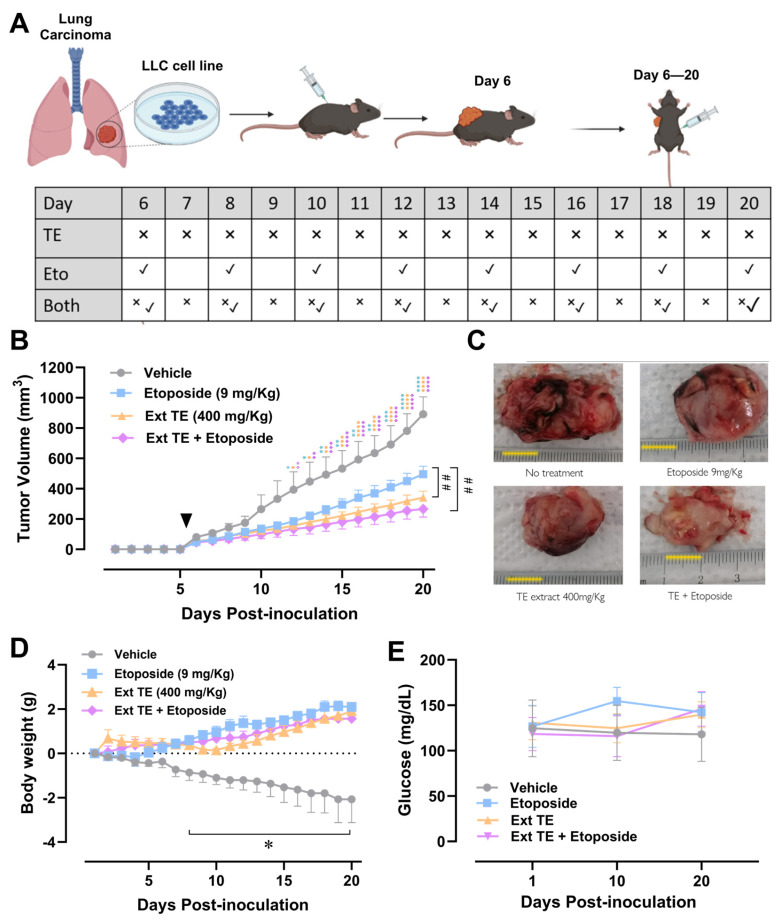
Floral extract decreases LLC tumor growth. (**A**) Murine model scheme for the treatment (**B**) Tumor growth. Tumor cells were implanted on the back of the mice; when the tumor was visible, the mice were randomized and separated into experimental groups. Tumor volume in mm^3^ was calculated using the Attia–Weiss formula, plotting the number of times the tumor size increased on the first day of treatment. The error bars indicate the mean ± SEM of 3 animals for each experimental group. Repeated measures Two-way ANOVA followed by Tukey post hoc, * *p* < 0.05, ** *p* < 0.01, *** *p* < 0.001, **** *p* < 0.0001 versus vehicle; ^##^ *p* < 0.01 between indicated groups. (**C**) Representative tumors obtained from the animals evaluated in (**B**), scale bar = 1 cm. (**D**) Changes in body weight. Changes in weight from day 1 post implantation were recorded. The change in weight concerning the first day of treatment, expressed in grams, was plotted. The error bars indicate the mean ± SEM of 3 animals for each experimental group. Repeated measures Two-way ANOVA followed by Tukey post hoc, * *p* < 0.05. (**E**) Changes in blood glucose levels. Three glucose measurements were taken, using a commercial glucometer on days 1, 10, and 20 post implantation. Error bars indicate the mean ± SEM of 3 animals for each experimental group. Repeated measures Two-way ANOVA followed by Tukey post hoc; there are no significant statistical differences.

The tumors were visible from day 6 post implantation, as shown with arrowhead in Figure 4B. From day 12 post implantation (day 6 of treatment), a significant difference in tumor growth began to be detected, showing growth inhibition in the groups treated with the *Tagetes erecta* extract, etoposide, and the combination.

At the end of the treatment (day 20 post implantation), it was observed that the 3 treated groups were able to delay tumor growth; significant differences between the group treated with etoposide and the group treated with the *T. erecta* extract were detected, which indicate that the administration of the *T. erecta* extract has a greater effect in slowing tumor growth compared to etoposide. Furthermore, the combination group (*T. erecta* extract + etoposide) had similar results to those of the extract, including a slight tendency to further decrease tumor volume compared to the group treated with *T. erecta*, indicating that the compounds present in the extract do not inhibit the effect of etoposide. In fact, the extract enhances the effect of etoposide, indicating that *Tagetes erecta* extract could have synergistic or additive effects with conventional therapy.

### 2.5. Tagetes erecta Extract Improves Body Weight Gain without Altering Glycemia in Tumor-Bearing Animal

In the control group with the greatest tumor growth, a drastic decrease in tumor weight is observed because of the deterioration of the health of the mice (Figure 4D), while in the treated groups with etoposide and extract, the weight was not only maintained during the first days but also increased throughout the experiment. The results began to be significant from day 8 post implantation, and weight gain was evident in all groups from day 14 post implantation.

To evaluate the metabolic control of the animals, glucose was evaluated at three points of experimental kinetics: on day 1 of implantation, day 10 post implantation, and the day of sacrifice (day 20). The sample was taken with a small cut in the tail of the mice using a commercial glucometer. As can be seen in Figure 4F, there were no significant changes in glucose levels throughout the treatment, which indicates that the *T. erecta* floral extract does not modify its metabolism.

### 2.6. T. erecta Extract Administration Decreases Proliferation and Tumor Viability

**Figure 5:**
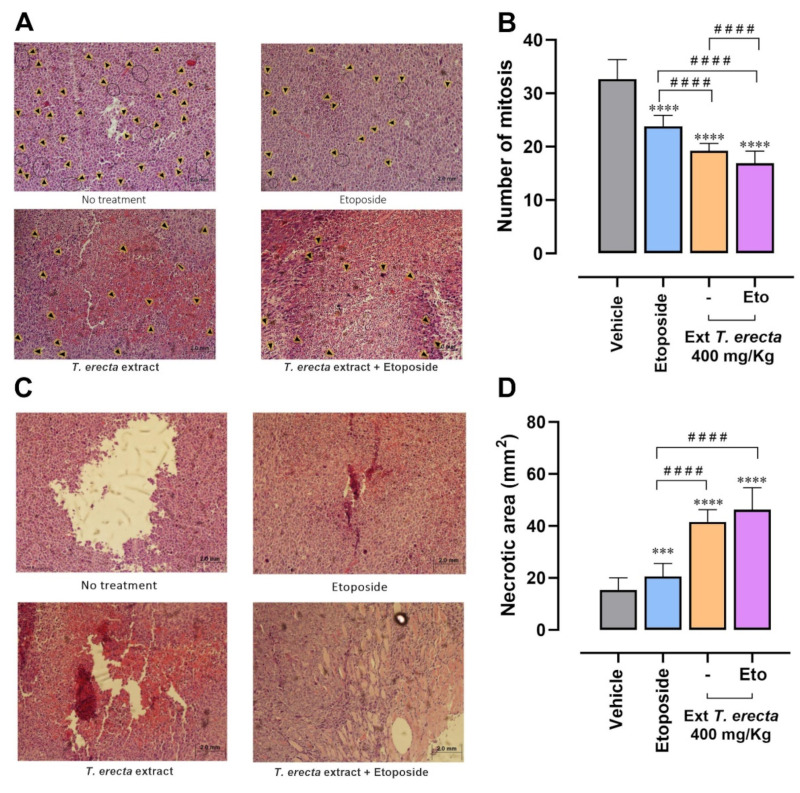
Floral extract of *T. erecta* decreases mitosis in LLC tumors. (**A**) Tumors were removed from treated and untreated mice, and then the mitoses were counted. In the photos, each arrowhead indicates that mitosis is taking place. (**B**) Mitosis counts in tumor sections. In hematoxylin eosin stained sections, the number of mitoses observed in 100 fields at 40× magnification was counted. The error bars indicate the mean ± SE of tumors for each experimental group. One-way ANOVA followed by Dunnett’s post hoc. **** *p* < 0.0001 versus vehicle; ^####^ *p* < 0.0001 versus etoposide. The result is represented as the number of mitoses. (**C**) Representative histological sections of the different treatments. Hematoxylin eosin stained sections from treated and untreated mice were analyzed. (**D**) Necrotic areas. In the stained sections of the mice sacrificed on day 20 post treatment with hematoxylin eosin, 50 fields per sample were analyzed. The error bars indicate the mean ± SE of 3 tumors for each experimental group. One-way ANOVA followed by Dunnett’s post hoc, *** *p* < 0.001, **** *p* < 0.0001 versus vehicle; ^####^ *p* < 0.0001 versus etoposide.

To assess whether the treatments decreased tumor proliferation, a mitotic count was performed via observing the histological sections under an optical microscope at 40× magnification. The mitotic count has a directly proportional relationship to tumor proliferation. In the following illustration, the mitoses found in the representative fields of the tumors extracted from treated and untreated mice are pointed with black arrow heads (Figure 5A). In the tumors extracted from the treated mice, the number of mitoses in the fields evaluated significantly decreased (Figure 5B). It was observed that the administration of the *T. erecta* extract, etoposide, and the combination significantly decreased cell proliferation. Furthermore, the combination of *T. erecta* extract with etoposide decreases proliferation more effectively than the individual administration of etoposide and the extract.

The stained histological sections were observed under an inverted microscope, where photographs of 50 fields per sample were taken. These images were analyzed with the Image J program to quantify the necrotic areas of the tumors. In Figure 5D, it is observed that the areas of necrosis in the tumors significantly increased in the treated groups with etoposide, extract, and the combination. The groups treated with *Tagetes erecta* extract had a greater increase of 40 mm^2^ in the necrotic areas, compared to the 20 mm^2^ obtained with the etoposide. There are significant differences between the groups treated with the extract, suggesting that tumor viability of the mice that received treatment with *Tagetes erecta* is lower than that of the mice treated only with etoposide.

### 2.7. GS–MS Study Showed the Presence of Various Bioactive Components in the Flowers of Tagetes erecta

**Figure 6:**
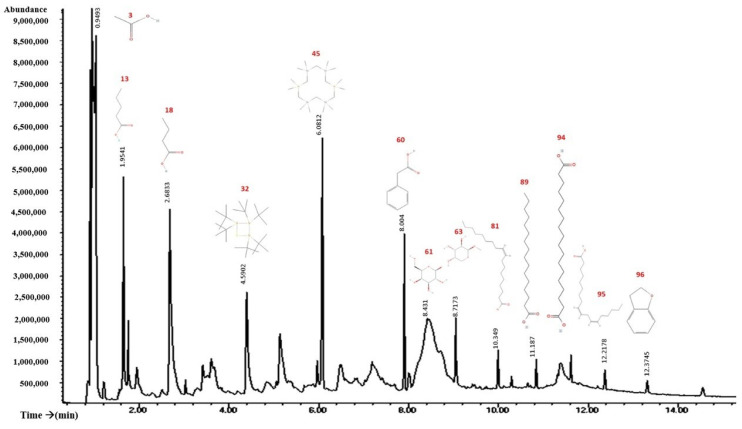
Chromatogram of *Tagetes erecta* flower extract.

The results of GC–MS analysis revealed the presence of various bioactive components in the flowers of *Tagetes erecta* (Figure 6 and Table 1). The major bioactive compounds detected in the flowers of *Tagetes erecta* were 2,3-dihydro benzofuran (coumaran), octadecanoic acid, benzenacetic acid, oleic acid, linoleic acid, acetic acid, methyl β-d-galactopyranoside, n-butyric acid, methyl β-l-arabinopyranoside, and n-hexadecanoic acid.

## 3. Discussion

Different types of *T. erecta* extracts have been evaluated in different cell lines in vitro. For example, Oliveira et al. [10] evaluated the impact of *T. erecta* extracts on five tumor cell lines and compared it with the effects of the extracts of other species. They found that the extract that had an effect in all the cell lines evaluated was *T. erecta;* the extract of flowers was also used and evaluated in the MCF-7 cells, where it had a cytotoxic effect of 30% [10], a result similar to that obtained in our experiment. In the reference article, the extract used was an essential oil. It is worth mentioning that the evaluations found that the essential oil extract had a greater effect on adenocarcinomas and melanomas, which are cells similar to those evaluated by our research group (small cell lung carcinoma). Furthermore, they used a non-tumor cell line V79 (hamster lung fibroblasts) as a cytotoxicity control, and the *T. erecta* extract did not have significant results in cytotoxicity evaluations. Other researchers, used the non-tumor cell line MDCK (Madin–Darby canine liver) and during their evaluations, no cytotoxic effects were observed at 24 and 48 h of treatment, which coincides with our in vitro results using the non-tumor cell line COS-1 [24].

We used the toxicity assay with *Artemia salina* instead of a model in higher animals; we did not observe signs of toxicity in the nauplii even after adding high concentrations of the extracts, which coincides with the results reported by Cui et al. [25], where they used a murine model in which concentrations of 200 and 400 mg/kg were administered orally in rats; no signs of toxicity were observed in rats and survival was 100 percent throughout the evaluation. Therefore, together with the in vitro evaluations, we can deduce that the administration of the *T. erecta* extract is safe.

There are two reports of in vivo models of the administration of *T. erecta* extracts in mice. Cui et al. [25] evaluated the administration of *T. erecta* essential oil in an acute model of induced gastric cancer. They found that the extract reverses the damage induced by cancer and prevents metastasis in other organs, so they concluded that the administration of essential oil can be used for the prevention and treatment of gastric cancer [25]. In addition, Barhoi et al. [6] carried out a model similar to ours; it is a xenograft of Ehlrich carcinoma cells (EACs) in albino mice. They used an aqueous extract of *T. erecta*, as in our experiment, and found that the administration of *T. erecta* maintains and increases the weight of the mice, in addition to reducing the size of the extracted tumors; this is similar to our result of tumor reduction in the tumors of mice that were treated.

In addition, the mitotic count decreases in the groups treated with *T. erecta* extract, which is associated with a decrease in cell proliferation and explains the decrease in tumor volume. The sum of these two evaluations allowed us to see the effect of the treatments on tumor viability, which refers to active and developing cells that can damage healthy tissue. The groups treated with *Tagetes erecta* and in combination with etoposide significantly decreased cell viability compared to the two controls used, obtaining more promising results than the approved drug used as a reference.

The phytochemical description of the extract developed by Barhoi et al. [6] has compounds that we also find in our extract; these compounds are hexadecanoic acid, linoleic acid, and 2,3-dihydrobenzofuran (coumaran), which have antitumor properties.

Many of the main compounds, even in isolation, have activities related to cancer treatment, ranging from the preventive effects of acetic acid, which can inhibit the generation of oxaloacetate [20], to the description of mechanisms including the inhibition of highly active enzymes in cancer cells, such as oleic acid which has the ability to inhibit telomerase, whose role is crucial in cancer cell immortalization [18]. Enzymes such as elastase and tyrosinase, which are capable of inhibiting prostate cancer cells, are also present in the root extracts of *T. erecta* [11]; these enzymes are known to be responsible for the transduction of adhesion and migration signals in tumor cells.

In addition, some of these compounds are found in other plant species that have anticancer activities, such as coumaran (2,3-Dihydro-benzofuran), which has anticancer activity in cancers such as prostate, kidney, breast, laryngeal, lung, colon, leukemia, and malignant melanoma [15]. It is known that derivatives of some acids were found to have inhibitory effects on cell proliferation and decrease metastasis in other types of cancer. Palmitic acid was studied and found that in cancer prostate, palmitic acid treatment induced G1 phase cell cycle arrest; in addition, it was found that it can inhibit prostate cancer cell metastasis [17]. Furthermore, a mechanistic study indicated that palmitic acid inhibited key molecules in the PI3K/Akt pathway to block prostate cancer proliferation and metastasis [23].

It is also known that n-butyric acid, present in our extract, is capable of inhibiting cell proliferation in colorectal cancer. In addition, Forte et al. [17] evaluated the derivatives of benzeneacetic acid in six tumor cell lines, where it was found to exert a cytotoxic effect. The sum of these compounds explains the effects that the flower extract of *T. erecta* had in the different stages and evaluations of this study. The evaluations carried out demonstrate the cytotoxic and anticancer potential of the *T. erecta* flower extract in vivo and in vitro evaluations.

## 4. Methods

### 4.1. Plant Extract

Fresh plants of TE were purchased from a local nursery in Zacatecas, Mexico, in November 2021. A taxonomist of the University Autonomous of Aguascalientes Mexico confirmed the identity of the plant material (Registry number TEUAA/nov21). Fresh flowers and leaves were mixed with 40% of ethanol/water and liquefied using an electric blender (T3L Tapisa, León, Mexico) to prepare the extract. Maceration remained for five days at room temperature. The solvent was changed twice, and the extracts were mixed, filtered, and placed in a rotatory evaporator (R100 Buchi, New Castle, DE, USA) to concentrate it. The concentrated extract was lyophilized (Free Zone 2.5L Labconco, Kansas City, MO, USA), and the dry powder was used in the biological assays.

### 4.2. Chromatography-Mass Spectrometry (GC/MS)

The TE flower extract was analyzed using gas chromatography coupled to mass (GS-MS) using an Agilent Technologies 5975C VL MSD, Santa Clara, CA, USA. The silica column used was Agilent 19091s-433E HP5MS, Santa Clara, CA, USA (30 m × 0.25 mm, 0.25 µm film thickness). The gas used was Helium, with a constant flow of 2.0 mL/min. Manual injection volume was 0.1 µL (Separation ratio: 33.4:1). The injector and ion source temperatures were set at 260 °C. The mass spectrum was taken at intervals of 2.5 min; the mass range was from 30 to 500 Da. Fragmentation was compared to the computer library (Adams, IL, USA, 2005).

### 4.3. Toxicity Test in Artemia Salina

*Artemia salina* cysts were used; 250 mg of them were incubated in 350 mL of water with 34 g/L of salinity at room temperature with lighting. The cysts were kept in suspension with continuous light aeration for 24 h until their hatching. The hatched cysts (nauplii) selected for the test were healthy and without malformations. A total of 10 nauplii were used per replicate; while 35 g/L of saline solution was used as the negative control, quillaja standard was used as the toxic control. These organisms are sensitive during their first 24–36 h of life; during this time, a series of concentrations of both TE extracts (50 mg/mL, 5 mg/mL, 50 µg/mL, 5 µg/mL, 50 ng/mL, and 5 ng/mL) were added. The experiment was then allowed to run for 24 h to make a count of the dead and alive Artemias.

### 4.4. Cell Cultures

Lewis lung carcinoma cells (LLC1 and ATCC CRL-1642), human breast carcinoma (MCF-7 and ATCC HTB-22), and monkey kidney fibroblasts (COS-1 and ATCC CRL-1650), obtained from the organization American Type Culture Collection (ATCC), Washington, DC, USA were cultured with a DMEM medium supplemented with 10% fetal bovine serum (Thermo Fisher Scientific, Gibco, Birmingham, MI, USA) and antibiotic (streptomycin-penicillin) at 37 °C with 5% CO_2_.

### 4.5. Cell Viability Assay

The cells were seeded in 24-well dishes for the treatments with *T. erecta* extracts. As a positive control, etoposide 50 µM (Tosuben, PiSA Laboratories, México City, Mexico) was used, which is a first-line drug used to treat lung carcinoma whose mechanism of action is to inhibit cellular topoisomerase. The cells were incubated for 24 h; then, a 10 µL sample was taken and mixed with 10 µL of trypan blue (Sigma-Aldrich, St. Louis, MO, USA). The resulting product was placed in a Neubauer chamber to proceed with the counting of the living (refractive) and dead (dyed blue) cells under an optical microscope in 40× objective.

### 4.6. Proliferation Assay

The cells treated with *T. erecta* extracts or etoposide (50 µM) were incubated for 24 h in a 24-well plate. Then, the culture medium was removed to separate the unbound cells, and the plate was washed 3 times with sterile PBS (phosphate-buffered saline), fixed with absolute methanol, and refrigerated at 4 °C for 20 min. Then, 300 µL of crystal violet (Sigma-Aldrich) solution was added, and it was incubated for 1 h at 4 °C. Next, the cells were washed with sterile PBS and 300 µL of acetic acid was added as a cellular lysis solution, 100 µL of each well in triplicate in a 96-well plate. They were then analyzed in a microplate reader (Fisher Scientific, Waltham, MA, USA) at an absorbance of 600 nm.

### 4.7. In Vivo Studies

#### 4.7.1. Animals

C57BL/6 mice of both sexes, from 45 days of age, weighing between 25 and 30 g, were kept in conditions of room temperature (22 °C), in a cycle of 12 h light/12 h darkness, and acclimatized for one week before handling. The animals were fed pellets with food and water ad libitum.

#### 4.7.2. Tumor Murine Model

Lewis lung carcinoma (LLC) cells were implanted subcutaneously into the back of 15 mice (1 × 10^6^ LLC cells/animal) using an ultrathin 1 mL syringe as previously reported [26]. Then, they were randomly divided into 4 experimental groups, and the mice were treated on day 6 post implantation. The control group was treated with injectable water. Positive control was injected intraperitoneally 3 times a week with 9 mg/Kg etoposide for 15 days. The experimental group was treated with 400 mg/Kg/day of the *Tagetes erecta* flower extract. The combination treatment group was administrated with 9 mg/Kg of etoposide 3 times a week and with 400 mg/Kg/day of extraction. The tumor volume of all experimental groups was followed from day 5 post implantation with an electronic vernier to calculate tumor volume (V) using the Attia and Weiss formula: V = 0.4 (Long diameter × Short diameter). Mice bearing LLC tumors were randomly divided among the experimental groups, treated, and sacrificed after 20 days of implantation using a CO_2_ chamber. Observe Figure 4A.

#### 4.7.3. Monitoring Changes in Body Weight and Systemic Glucose

Mice were weighed daily from the day of implantation (day 1) until the day of sacrifice, and changes in body weight were reported for each day. Three glucose measurements were taken—on the day of implantation, on day 10, and on the day of sacrifice—with an Accu-check^®^ Performa glucometer (Accu-Check, Corydon, IN, USA).

### 4.8. Ex Vivo Model

Biopsies of the excised tumors were taken; to subsequently fix them in a 10% formalin solution, the samples were submerged for a period of 48 h in the solution, and then the tissue was embedded in paraffin. Once the paraffin block was obtained, cuts were carried out in a microtome, and the cuts obtained were spread and adhered to slides; then, they were deposited onto the surface of the liquid contained in a container called a “flotation bath”. In the staining procedure, hematoxylin eosin was added; in this staining process, the nuclei are stained with hematoxylin, and then eosin is responsible for coloring the cytoplasm. The nuclei stain appears to be purple, while the cytoplasm and extracellular material stain appear in various shades of pink.

### 4.9. Histopathological Evaluation of Mitotic Cells and Necrotic Areas

For the analysis of mitotic cells, 100 fields were considered in the total samples per group, and an optical microscope with 40× magnification was used. An inverted microscope was used for the analysis of necrotic areas; they were photographed and analyzed with the Image J program. In total, 50 fields per sample were considered.

### 4.10. Statistical Analysis

To demonstrate differences between more than two groups, a one-way Analysis of Variance (ANOVA) and Dunnett’s post hoc were used. Recording of tumor volume during days was analyzed using repeated measures two-way ANOVA with Sidak’s or Tukey post-hoc. Results were expressed as mean ± standard error deviation or standard deviation as indicated in the legends of the figures. The data were considered statistically different when *p* < 0.05.

## 5. Conclusions

The hydroalcoholic extract of *T. erecta* flowers and leaves has a cytotoxic effect on Lewis lung carcinoma cells. Particularly, the flower extract showed high effectiveness in lung cancer cells, and interestingly, had selectivity to cancer cell lines without effects on non-cancer cells. The present study indicates that the *T. erecta* flower extract inhibits tumor progression and increases its effectiveness in combination with etoposide. The results contribute to the evidence that the bioactive compounds present in the hydroalcoholic extract of *T. erecta* have therapeutic potential against lung cancer. The compounds found in the floral extract to which the antitumor effect is attributed are coumaran, octadecanoic acid, benzeneacetic acid, oleic acid, linoleic acid, acetic acid, methyl-β-d-galactopyranoside, n-butyric acid and n-hexadecanoic acid.

## Figures and Tables

**Table 1:** Main compounds found in chromatography-mass spectrometry (GC/MS).

Beak	Holding Time	% Area	Compound	Effect Reported in Cancer	Refs
96	12.3745	21.13	2,3-Dihydro-benzofuran (coumaran)	Anticancer properties	[15]
94	11.8127	15.32	Octadecanoic acid	Nanocarrier for anticancer drugs	[16]
60	8.004	12.1	Benzeneacetic acid	Antiproliferative effect	[17]
81	10.3487	11.23	Oleic acid	Telomerase inhibitor	[18]
95	12.2178	11.2	Linoleic acid	Anticarcinogenic	[19]
3	0.9493	9.49	Acetic acid	Preventive function against cancer	[20]
63	8.7173	6.96	Methyl-β-d-galactopyranoside	Inhibits the growth of Ehrlich ascites carcinoma	[21]
18	2.6833	6.61	n-butyric acid	Colorectal cancer treatment	[22]
61	8.431	6.34	Methyl β-l-arabinopyranoside	Unknown	
89	11.186	5.79	n-hexadecanoic acid	Inhibits proliferation and metastasis in prostate cancer	[23]

## Data Availability

Not applicable.

## Sample Availability

Samples of the compounds are not available from the authors.
